# Influence of short‐term training on functional capacity and (anti‐)inflammatory immune signalling in acute hospitalization

**DOI:** 10.1002/jcsm.12582

**Published:** 2020-06-08

**Authors:** Robinson Ramírez‐Vélez, Nicolás Martínez‐Velilla, Joaquín Fernández‐Irigoyen, Enrique Santamaría, Sara Palomino‐Echeverría, Mikel Izquierdo

**Affiliations:** ^1^ Navarrabiomed Complejo Hospitalario de Navarra (CHN)‐Universidad Pública de Navarra (UPNA), IdiSNA Pamplona Spain; ^2^ CIBER of Frailty and Healthy Aging (CIBERFES) Instituto de Salud Carlos III Madrid Spain; ^3^ Proteored‐Institute of Health Carlos III (ISCIII), Clinical Neuroproteomics Unit, Navarrabiomed, Navarra Health Department Public University of Navarra, Navarra Institute for Health Research (IdiSNA) Pamplona 31008 Spain

## Introduction

It has been shown that 7 days of in‐hospital inactivity induces a rapid decline (>10%) in total lean leg mass in healthy older adults,^1^ and loss of muscle mass has been associated with a lower likelihood of survival after hospitalization in older patients.^2^ In this regard, chronic, low‐grade inflammation is a hallmark of aging and is associated with changes in body composition and declining physical function, making older adults even more vulnerable to the negative impact of hospitalization.^2^ The release of muscle‐derived myokines is responsible for many of the beneficial effects of exercise in older adults, particularly by promoting a healthy anti‐inflammatory milieu^3^ and may thus complement the anti‐inflammatory and analgesic actions of medications.

To investigate the influence of exercise on inflammatory signalling,^3^ we performed cytokine array profiling in human serum to identify inflammatory cytokines produced after a 3 day in‐hospital intervention including individualized moderate‐intensity resistance, balance, and walking exercises vs. medical usual‐care for acute hospitalization in very elderly patients.

The study was conducted at the Department of Geriatrics, *Complejo Hospitalario de Navarra* (Research Ethics Committee ID Pyto2018/7, No. 264, dated 15 April 2018). All patients or their legal representatives provided written consent. The usual‐care group (*n* = 20) received habitual hospital care, which included physical rehabilitation when needed. For the intervention group (*n* = 18), exercise training was programmed in two daily sessions (morning and evening) of 20 min duration during five to seven consecutive days (including weekends) supervised by a qualified fitness specialist.^4^

Quantification of 80 analytes in EDTA plasma samples was performed at Cytokine Array–Human Cytokine Antibody Array (ABCAM Membrane, 80 Targets # ab133998) (Myriad RBM, Austin, USA). Medical and functional characteristics in each group were reported as the estimated margin of the mean, as assessed by 95% confidence intervals using a two‐factor [group (control or exercise) × time (baseline and after intervention)] repeated‐measures analysis of covariance (ANCOVA) with the pre‐test used as a covariate in the model.^3^ We used relative treatment effect (RTE) for exercise cytokine response. RTE can be briefly interpreted as the effects through the normalized data distribution (ranges from 0 to 1). Correlation coefficients (*r*) are shown as the Pearson index on training responsiveness (Δ).

**FIGURE 1:**
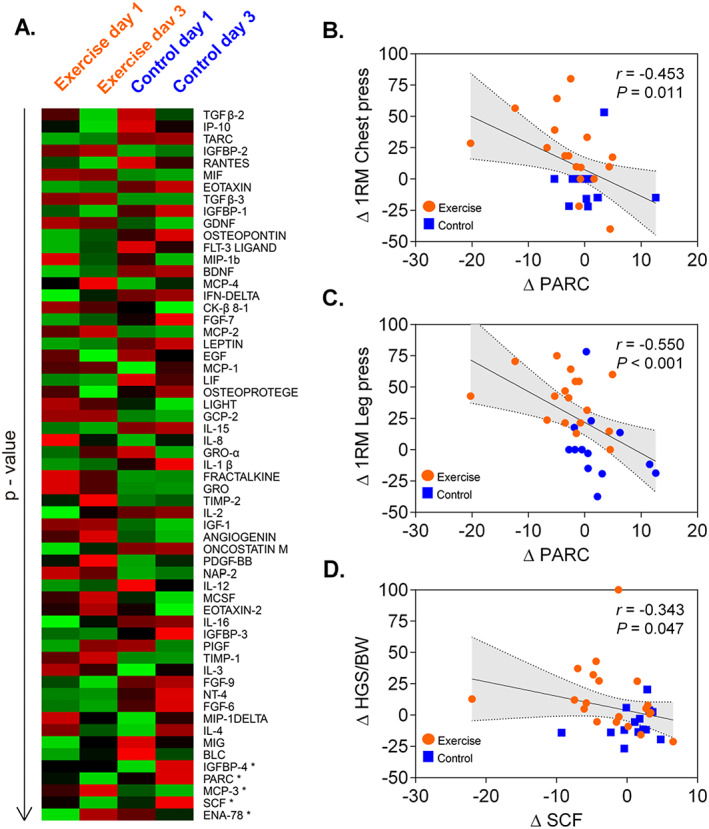
(A–D) Hierarchical cluster analysis and cytokine response of significant variables with main effect time point and interaction effect at each time point between exercised and control individuals using Ward's method. Differences are represented by the computed relative treatment effect (RTE). Red colour represents higher RTES, and green lower RTEs. *P < 0.05; *P*‐value indicates significant interaction effects between group and time. LIGAND for the receptor‐type protein‐tyrosine kinase KIT. ENA‐78/CXCL5, C‐X‐C motif chemokine 5; IGFBP‐4, IGF‐binding proteins; MCP‐3, monocyte chemotactic protein 3; SCF/KITLG, kit ligand; PARC/CCL18, C‐C motif chemokine 18.

No adverse effects associated with the intervention were recorded, and no patient had to interrupt the intervention or had their hospital stay modified because of it. ANCOVA models revealed a significant group × time interaction in handgrip strength (+2.5 kg), one repetition maximum (1RM) chest press (+5.5 kg), short physical performance battery score (+1.0), and gait speed (−0.11 m/s) (all *P* < 0.01) (*Table* 1). A total of 80 human proteins were performed at Cytokine Array, and 59 were quantified with the ImageQuant ECL system. The levels of five inflammatory mediator cytokines differed between groups (*Figure* 1): MCP‐3 and ENA‐78 levels were higher in the exercise group, whereas Insulin‐like growth factor binding protein‐4 (IGFBP‐4), C‐C motif chemokine 18 (PARC), and stem cell factor (also known as SCF, KIT‐ligand, KL, or SCF) levels were lower. Correlation analysis of the full sample revealed a negative relationship between ΔPARC levels and Δ1RM chest press (*r* = −0.453, *P* = 0.011) and Δ1RM leg press (*r* = −0.550, *P* < 0.001). Furthermore, a significant negative correlation was observed between ΔSCF levels and 1RM chest press (*r* = −0.399, *P* = 0.028) and Δhandgrip strength/body weight (*r* = −0.343 *P* = 0.047) from baseline to exercise.

**TABLE 1:** Results of endpoints by group

Variable	Exercise group	Control group	Between‐group difference (95% CI)	*P*‐value
SPPB (balance, gait ability, leg strength), score^a^	1.35 (2.23 to 0.46)	0.33 (−1.33 to 0.66)	1.02 (−0.20 to 2.24)	0.098
Handgrip strength, kg	1.51 (2.91 to 0.10)	−0.99 (−0.33 to 2.32)	2.50 (0.73 to 4.28)	0.007
Handgrip strength, kg/body weight, kg	0.01 (−0.00 to 0.03)	−0.02 (−0.04 to 3.08)	0.04 (0.01 to 0.06)	0.013
Gait speed, m/s	−0.11 (−0.18 to −0.04)	0.01 (−0.10 to 0.09)	−0.10 (−0.21 to 0.00)	0.061
1RM chest press, kg	3.63 (6.99 to 0.27)	−1.91 (−0.78 to 4.61)	5.55 (1.70 to 9.40)	0.006
1RM chest press, kg/body weight, kg	0.02 (0.01 to 0.06)	0.05 (0.10 to 0.01)	0.08 (0.03 to 0.14)	<0.001
1RM leg press, kg	17.21 (22.44 to 11.97)	−0.57 (−11.05 to 12.20)	17.78 (7.80 to 27.76)	<0.001
1RM leg press, kg/body weight, kg	0.26 (0.32 to 0.18)	0.005 (−0.16 to 0.17)	0.29 (0.14 to 0.43)	<0.001
Mini‐Mental State Examination, score^b^	1.31 (−2.69 to 0.06)	0.42 (−2.26 to 1.40)	0.89 (−1.10 to 2.88)	0.370
Barthel index (ADLs), score^c^	3.00 (−10.57 to 4.56)	1.34 (−9.18 to 9.08)	3.00 (−7.79 to 13.79)	0.577
Depression (GDS), score^d^	0.89 (−0.08 to 1.87)	−0.06 (−1.16 to 1.03)	−0.96 (−2.26 to 0.34)	0.146

Abbreviations*:* 1RM, one repetition maximum; ADLs, activities of daily living; SPPB, Short Physical Performance Battery.

a The SPPB scale ranges from 0 (worst) to 12 (best).

b The Mini‐Mental State Examination ranges from 0 (worst) to 30 (best).

c The Barthel index ranges from 0 (severe functional dependence) to 100 (functional independence).

d The Yesavage Geriatric Depression Scale ranges from 0 (best) to 15 (worst).

## Discussion

Physical exercise training, tailored to the disease and to the specific needs of the patient, has become a cornerstone of modern therapeutic interventions for many chronic pathological conditions.^5^ A 3 day in‐hospital intervention modifies the serum levels of MCP‐3 (increased), PARC (decreased), and ENA‐78 (increased), which are all essential to promote myoblast proliferation, physiological homing of mononuclear blood cells, and inflammatory responses.^6^ Higher PARC levels have recently been related to a higher degree of cardiac dysfunction and a lower exercise capacity.^7^ We also found that IGFBP‐4 and SCF levels were reduced after the in‐hospital intervention. Elevated plasma levels of SCF and its variants are associated with inflammatory bowel disease and germ cell tumours respectively, whereas IGFBP family members are linked to breast, endometrial, colon, and skin cancers.^6^ Our results might be a first important step to better understand the potential role of inflammation in short‐term adaptation to 3 day in‐hospital intervention.

Our findings shed new light on the development of in‐hospital strategies to prevent/reverse the functional decline during acute hospitalization and for health status monitoring.

## Author Contributions

Ramírez‐Vélez had full access to all of the data in the study and takes responsibility for the integrity of the data and the accuracy of the data analysis. Concept and design were performed by Martínez‐Velilla, Izquierdo, and Ramírez‐Vélez. Acquisition, analysis, or interpretation of data was performed by all authors. Drafting of the manuscript was performed by Ramírez‐Vélez and Palomino. Critical revision of the manuscript for important intellectual content was carried out by all authors. Statistical analysis was performed by Palomino, Ramírez‐Vélez, Fernández‐Irigoyen, and Santamaría. Izquierdo obtained the funding. Supervision was conducted by Izquierdo, Martínez‐Velilla, and Ramirez‐Velez.

## Conflict of Interest

No disclosures were reported.

## Role of the Funder/Sponsor

The funder had no role in the design and conduct of the study; collection, management, analysis, and interpretation of the data; preparation, review, or approval of the manuscript; and decision to submit the manuscript for publication.
